# The Psychological Impact of the Coronavirus Disease 2019 Pandemic on Pregnant Women in China

**DOI:** 10.3389/fpsyt.2021.628835

**Published:** 2021-07-02

**Authors:** Zheng Zheng, Ruoxi Zhang, Tao Liu, Pei Cheng, Yanhong Zhou, Weicong Lu, Guiyun Xu, Kwok-Fai So, Kangguang Lin

**Affiliations:** ^1^Department of Obstetrics, Guangzhou Women and Children's Medical Center, Guangzhou, China; ^2^Department of Affective Disorders, The Affiliated Brain Hospital of Guangzhou Medical University (Guangzhou Huiai Hospital), Guangzhou, China; ^3^Guangdong-Hong Kong-Macau Institute of Central Nervous System Regeneration, Jinan University, Guangzhou, China; ^4^The State Key Laboratory of Brain and Cognitive Sciences, Department of Ophthalmology, University of Hong Kong, Hong Kong, China

**Keywords:** anxiety, COVID-19, depression, pregnant women, stress

## Abstract

**Background:** The coronavirus disease 2019 (COVID-19) pandemic has been reported to have negative psychological impact on mental health. Nonetheless, there are few studies investigating the impacts on pregnant women. This study investigated the psychological impact of COVID-19 pandemic on pregnant women, and the associated risk factors that moderated this impact.

**Methods and Materials:** A total of 2,798 pregnant participants were recruited from the Guangzhou Women and Children's Medical Center. The Patient Health Questionnaire-9 (PHQ-9), Generalized Anxiety Disorder-7 (GAD-7) and Insomnia Severity Index (ISI) were used to assess depression, generalized anxiety disorder and insomnia, respectively, during and before the COVID-19 pandemic. The Impact of Event Scale-Revised (IES-R) was used to assess psychological stress during the COVID-19 pandemic.

**Results:** During the COVID-19 pandemic, over one third of pregnant participants reported mild depression, around 20% experienced mild generalized anxiety, about one third reported problems with sleeping, and more than 15% felt mild psychological stress. The occurrence of psychological problems was significantly higher during the COVID-19 pandemic when compared to before the outbreak. The previously described pattern that pregnant women in the first trimester are more likely to report depression, and those in the third trimester are more likely to report insomnia and psychological stress, was also recognized in our study population. Mental health issues existing before the outbreak were risk factors, while family support was a protective factor in the occurrence of the measured mental health problems during the COVID-19 pandemic.

**Conclusion:** Our data suggest pregnant women's mental health is inevitably affected during the COVID-19 pandemic. Pregnant women in the first and third trimester and those who experienced mental issues before the outbreak may be particularly affected.

## Introduction

The coronavirus disease 2019 (COVID-19) pandemic is one of the most devastating infectious diseases in recent history, with hundred millions of infected people and over two hundred thousands of deaths (1). This global public health emergency has a profound effect on many aspects of the society (2).

Pregnant women are commonly afflicted by mood symptoms (3, 4). Previous studies show that infectious disease outbreaks increase risk of anxiety and negative feelings among pregnant women (5, 6). A study in China reported that the rate of depression was significantly higher among pregnant women after the declaration of COVID-19 epidemic than before the COVID-19 epidemic (7). During the current COVID-19 pandemic, a Canadian study suggested that 37% pregnant women reported clinically significant levels of depression and 57% pregnant women reported clinically significant levels of anxiety (8). A recent meta-analysis showed that the prevalence rates of depression and anxiety among pregnant women during COVID-19 pandemic were 31 and 37%, respectively (9).

Prenatal mental health is crucial to physical and psychological health of the mother and the fetus. Some prospective studies demonstrated that serious perceived stress was significantly associated with shorter gestational time, lower birthweight, lower Apgar scores and higher rates of complications (10). Pregnant women infected with COVID-19 were more likely to have low birth weight, preterm delivery, and perinatal death (11, 12). Moreover, prenatal stress, depression and anxiety are reported to have negative effects on the neurodevelopment, cognition and temperament of the newborns (13).

The prenatal period could be a more vulnerable time as the COVID-19 pandemic produces additional stress on pregnant women (14). The stress of pregnant women increased as a result of uncertainties in antenatal care, exposure risk for both mother and baby, and lack of support network due to quarantine at home and movement restriction (15). The levels of psychological distress may change across different pregnancy trimesters. A study suggested that the level of depression decreased from the first to the third trimester, while anxiety symptoms manifested in a U pattern (16). The levels of prenatal stress symptoms in early and later time were higher than that in the middle time (17). Furthermore, pregnant women with a pre-existing mental illness were more vulnerable compared to those without (18). The COVID-19 pandemic may result in postponing or cancelation of consultations and discontinuation of medications.

Given the above considerations, we in this study investigated the occurrences of depression, anxiety, insomnia and psychological stress in pregnant women during this COVID-19 pandemic. We hypothesized that pregnant women may suffer more mental health issues during the first trimester compared to the third trimester. In addition, we investigated risk factors that affected depression, anxiety, insomnia and psychological stress in pregnant women during theCOVID-19 pandemic.

## Methods

### Participants

Pregnant women who received prenatal examinations in the Guangzhou Women and Children Medical Care Center in Guangzhou, China were recruited from March 7 to April 30, 2020 during the COVID-19 pandemic. Inclusion criteria were pregnant women aged 18–50 years. The study was approved by the Institutional Review Board of Guangzhou Women and Children Medical Care Center. All participants gave informed consent.

### Measurements

#### Socio-Demographic Characteristics

Socio-demographic data were self-reported, including history of mental illness and family support. We converted socio-demographic variables (except age) into binary variables. Participants were of Han and minority ethnicities. Relationship status was dichotomized as “married” and “not married” (single, divorced and widow). Education status was divided into lower education status (senior middle school and below) and higher education status (college degree and above). Occupation was dichotomized as “employment” (civil servant, enterprise employee, freelance and medical staff) and “unemployment” (homemaker and the unemployed). Family support was recorded as “less support” and “more support.”

#### Mental Health Assessment

The Patient Health Questionnaire 9 (PHQ-9) (19), Generalized anxiety disorder 7 (GAD-7) (20), and the Insomnia Severity Index (ISI) (21)were used to assess depression, generalized anxiety, and insomnia, respectively. The Impact of Event Scale-Revised (IES-R) (22) was administered to evaluate psychological stress status. Participants were asked to rate their mental status during the COVID-19 pandemic, and were asked to retrospectively rate the status of the past 2 weeks or 1 month before the COVID-19 epidemic was declared by the Chinese government on January 20, 2020.Mild depression and mild generalized anxiety were, respectively, defined by a score of 5 or above on the PHQ-9 and GAD-7 (23, 24). A cut-off of 8 points was used on the ISI to assess mild insomnia (21). A cut-off of 24 points was used on the IES-R to assess mild psychological stress symptom (25, 26).

### Statistical Analysis

SPSS 26.0 software (SPSS Inc., Chicago, IL, USA) was used for performing statistical analyses. All tests were two-tailed, with a significance level of *p* < 0.05. McNemar's test and Wilcoxon signed-rank test were used to compare the scores of depression, generalized anxiety, and insomnia in pregnant women before and during the COVID-19 pandemic. The occurrences and total scores of depression, generalized anxiety, insomnia and psychological stress were compared among different pregnancy trimesters using Chi-square test and Kruskal-Wallis H test, respectively. Bonferroni correction was applied for multiple comparisons. Binary logistic regressions were conducted to investigate the risk factors for depression, generalized anxiety, insomnia and psychological stress during COVID-19 pandemic. The analysis models included the following factors: age, relationship status, education, occupation, pregnancy trimesters, history of mental illness, family support, scores of PHQ-9, GAD-7, and ISI before the COVID-19 outbreak.

## Results

### Socio-Demographic Characteristics

Two thousand nine hundred eighty-five pregnant women were invited to participate in the study, and 187 refused to participate. The resultant sample included 2,798 pregnant women, of whom 2,273 finished both the surveys that rated their psychological status during and before the COVID-19 pandemic. One thousand two hundred twelve (43.3%) participants were aged 26–30 years old and 1,168 (41.7%) participants were aged 31–40 years old. Han ethnicity accounted for 96.7% of the sample. Two thousand seven hundred five (96.7%) participants were married. Six hundred seventy-nine (24.3%) pregnant women were at lower education status, while 2,119 (75.7%) were at higher education status. Two thousand two hundred twenty-five (79.5%) participants were at work, while 573 (20.5%) were unemployed. Twenty-two (0.8%) participants had a history of mental illness. Eighty-seven (3.1%) had less family support and 2,711 (96.9) had more family support. Moreover, participants were divided into three groups based on their gestation age, which were the first trimester (<14 weeks), second trimester (14–28 weeks) and third trimester (more than 28 weeks). 13.8% of the participants were in the first trimester, 27.6% in the second trimester, and 58.6% were in the third trimester. The socio-demographic characteristics of pregnant women in different trimesters are shown in Table 1. Chi-square test revealed that women in different trimesters reported statistically different relationship status (χ^2^ = 40.57, *P* < 0.01).

**Table 1:** Socio-demographic characteristics of pregnant women among pregnancy trimester during the COVID-19 pandemic.

		**First trimester *n* (%) (*n* = 386) (A)**	**Second trimester *n* (%) (*n* = 773) (B)**	**Third trimester *n* (%) (*n* = 1,639) (C)**	***P*-value**	***Post hoc*^**a**^**
Age	18–25	65 (16.8)	108 (14.0)	220 (13.4)	0.34	
	26–30	177 (45.9)	333 (43.1)	702 (42.8)		
	31–40	142 (36.8)	324 (41.9)	702 (42.8)		
	41–50	2 (0.5)	8 (1.0)	15 (0.9)		
Ethnicity	Han	375 (97.2)	742 (96.0)	1,588 (96.9)	0.44	
	Minority	11 (2.8)	31 (4.0)	51 (3.1)		
Relationship status	Married	356 (13.2)	738 (27.3)	1,611 (59.6)	<0.01	A, B < C
	Not married	30 (32.3)	35 (37.6)	28 (30.1)		
Education	Lower education status	95 (14.0)	183 (27.0)	401 (59.1)	0.90	
	Higher education status	291 (13.7)	590 (27.8)	1,238 (58.4)		
Occupation	Employment	371 (13.6)	757 (27.9)	1,590 (58.5)	0.19	
	Unemployment	15 (18.8)	16 (20.0)	49 (61.3)		
History of mental illness	Yes	2 (0.5)	10 (1.3)	9 (0.5)	0.12	
	No	384 (99.5)	763 (98.7)	1,630 (99.5)		
Family support	Less support	10 (2.6)	24 (3.1)	53 (3.2)	0.81	
	More support	376 (97.4)	749 (96.9)	1,586 (96.8)		

a *Bonferroni correction*.

### Psychological Status During the COVID-19 Pandemic and Before the Outbreak

Overall, 802 (35.3%), 433 (19.0%), 673 (29.6%) and 378 (15.2%) participants reported mild forms of depression, generalized anxiety, insomnia and psychological stress during the COVID-19 pandemic, respectively. Table 2 shows that pregnant women reported significantly higher occurrence of depression (35.3 vs. 22.4%, χ^2^ = 247.61, *P* < 0.01), generalized anxiety (19.0 vs. 13.9%, χ^2^ = 67.77, *P* < 0.01), and insomnia (29.6 vs. 23.9%, χ^2^ = 76.92, *P* < 0.01) during the COVID-19 pandemic than those before the COVID-19 outbreak. Table 3 displays that the total scores of PHQ-9, GAD-7 and ISI were significantly higher during COVID-19 pandemic than those before the outbreak (PHQ-9: 3.70 ± 3.84 vs. 2.43 ± 3.37, *Z* = −25.63, *P* < 0.01; GAD-7: 2.03 ± 3.20 vs. 1.45 ± 2.81, *Z* = −15.27, *P* < 0.01; ISI: 5.54 ± 4.62 vs. 4.63 ± 4.31, *Z* = −21.09, *P* < 0.01).

**Table 2:** Differences of the occurrence of mild forms of depression, generalized anxiety and insomnia during the COVID-19 pandemic and before the outbreak.

	**Before COVID-19 pandemic *n* (%) (*n* = 2,273)**	**During COVID-19 pandemic *n* (%) (*n* = 2,273)**	**χ^2^**	***P-*value**
Mild depression (PHQ-9 ≥ 5)	510 (22.4)	802 (35.3)	247.61	<0.01
Mild generalized anxiety (GAD-7 ≥ 5)	315 (13.9)	433 (19.0)	67.77	<0.01
Mild insomnia (ISI ≥ 8)	544 (23.9)	673 (29.6)	76.92	<0.01

**Table 3:** Differences of the total score of PHQ-9, GAD-7 and ISI during the COVID-19 pandemic and before the outbreak.

	**Before COVID-19 pandemic (Mean ± SD) (*n* = 2,273)**	**During COVID-19 pandemic (Mean ± SD) (*n* = 2,273)**	**Mean difference (95%CI)**	***Z***	***P-*value**
PHQ-9	2.43 ± 3.37	3.70 ± 3.84	1.26 (1.17–1.36)	−25.63	<0.01
GAD-7	1.45 ± 2.81	2.03 ± 3.20	0.58 (0.49–0.66)	−15.27	<0.01
ISI	4.63 ± 4.31	5.54 ± 4.62	0.08 (0.06–0.09)	−21.09	<0.01

### Psychological Status During the COVID-19 Pandemic Among Pregnancy Trimesters

Table 4 reports that the rates of the occurrence of mild forms of depression, insomnia and psychological stress during the COVID-19 pandemic across pregnancy trimesters were statistically different (depression: χ^2^ = 9.04, *P* = 0.01; insomnia: χ^2^ = 35.08, *P* < 0.01; psychological stress: χ^2^ = 12.85, *P* < 0.01). Further analysis revealed that pregnant women in the first trimester were more likely to experience depression than those in the third trimester (adjusted *P* < 0.01). The occurrence of insomnia was higher in the third trimester when compared with the occurrence in the first and second trimesters (adjusted *Ps* < 0.01), and pregnant women showed significantly higher occurrence of psychological stress in the third trimester than those in the first and second trimesters (adjusted *Ps* < 0.01).

**Table 4:** The occurrence of mild forms of depression, generalized anxiety, insomnia and psychological stress during the COVID-19 pandemic among pregnancy trimesters.

	**First trimester *n* (%) (*n* = 386) (A)**	**Second trimester *n* (%) (*n* = 773) (B)**	**Third trimester *n* (%) (*n* = 1,639) (C)**	**χ^2^**	***P-*value**	***Post hoc*^**a**^**
Mild depression (PHQ-9 ≥ 5)	162 (42.0)	278 (36.0)	555 (33.9)	9.04	0.01	A > C
Generalized anxiety (GAD-7 ≥ 5)	70 (18.1)	145 (18.8)	331 (20.2)	1.23	0.54	
Mild insomnia (ISI ≥ 8)	84 (21.8)	196 (25.4)	563 (34.4)	35.08	<0.01	A, B < C
Mild psychological stress (IES-R ≥ 24)	43 (11.1)	100 (12.9)	281 (17.1)	12.85	<0.01	A, B < C

a *Bonferroni correction*.

In parallel, Table 5 shows that significant differences among pregnancy trimesters were also found for the total scores of PHQ-9, GAD-7, ISI and IES-R during the COVID-19 pandemic (PHQ-9: *H* = 8.62, *P* = 0.01; GAD-7: *H* = 7.26, *P* = 0.03; ISI: *H* = 64.63, *P* < 0.01; IES-R: *H* = 44.94, *P* < 0.01). After Bonferroni correction, the total scores of PHQ-9 in the first trimester were higher when compared with the second and third trimesters (adjusted *Ps* < 0.01). The total scores of GAD-7 in the third trimester were higher than those in the first trimester (adjusted *P* < 0.01). The total scores of ISI and IES-R increased throughout the first, second and third pregnancy trimesters (adjusted *Ps* < 0.01).

**Table 5:** The total score of PHQ-9, GAD-7, ISI and IES-R during the COVID-19 pandemic among pregnancy trimesters.

	**First trimester (Mean ± SD) (*n* = 386) (A)**	**Second trimester (Mean ± SD) (*n* = 773) (B)**	**Third trimester (Mean ± SD) (*n* = 1,639) (C)**	**H**	***P*-value**	***Post hoc*^**a**^**
PHQ-9	4.29 ± 4.06	3.60 ± 3.60	3.69 ± 3.91	8.62	0.01	A > B, C
GAD-7	1.80 ± 2.90	2.03 ± 3.24	2.15 ± 3.22	7.26	0.03	A < C
ISI	4.47 ± 4.30	5.10 ± 4.37	6.21 ± 4.84	64.63	<0.01	A < B < C
IES-R	7.95 ± 11.10	10.00 ± 11.97	11.65 ± 12.74	44.94	<0.01	A < B < C

a *Bonferroni correction*.

### Logistic Regression Analysis

Binary logistic regression models were conducted to examine the factors that affect the occurrence of the measured clinical conditions. The adjusted models included factors including age, relationship status, education, occupation, pregnancy trimesters, history of mental illness, family support, and scores of mental health before the outbreak. Overall, the logistic regression models showed that the scores of PHQ-9, GAD-7 and ISI before the outbreak were positively correlated with the occurrence of depression, generalized anxiety and insomnia during the COVID-19 pandemic (depression: OR = 2.20, 95%CI: 2.07–2.35, *P* < 0.01; generalized anxiety: OR = 2.08, 95%CI: 1.95–2.21, *P* < 0.01; insomnia: OR = 2.11, 95%CI: 1.98–2.25, *P* < 0.01). More family support was significantly associated with reduced occurrences of the measured mental health problems (depression: OR = 0.28, 95%CI: 0.13–0.59 *P* < 0.01; generalized anxiety: OR = 0.22, 95%CI: 0.11–0.43, *P* < 0.01; psychological stress: OR = 0.44, 95%CI: 0.27–0.72, *P* < 0.01), indicating that family support was a protective factor.

Furthermore, the second and third trimester were associated with reduced occurrence of depression compared with the first trimester (the second trimester: OR = 0.43, 95%CI: 0.29–0.62, *P* < 0.01; the third trimester: OR = 0.44, 95%CI: 0.31–0.62, *P* < 0.01). Higher risk of psychological stress was associated with the third trimester compared with the first trimester (OR = 1.62, 95%CI: 1.14–2.28, *P* < 0.01). Pregnant women aged 26–30 years old, and those aged 31 to 40 years old, were more likely to report clinically significant psychological stress compared with pregnant women aged 18–25 years old (aged 26–30: OR = 2.06, 95%CI: 1.37–3.10, *P* < 0.01; aged 31–40: OR = 2.12, 95%CI: 1.41–3.20, *P* < 0.01).

## Discussion

To the best of our knowledge, although there have been some studies investigating the psychological well-being of pregnant women during the COVID-19 pandemic, few studies have considered the impact of the women's mental conditions before the outbreak.

Significantly higher rates of depression and insomnia among pregnant women were found during the COVID-19 pandemic. A recent study in China reported that 29.6% of pregnant women showed depressive symptoms after the COVID-19 outbreak (7), and a study in the U.S. suggested that the rate of depression was 36.4% during the COVID-19 pandemic (27). A recent meta-analysis indicated that the prevalence of depression was 31% (9), which was similar with our results. The occurrence of insomnia was higher than that (2.6%) in another Chinese study conducted in Beijing (28). Strict epidemic prevention policies were applied, such as reducing unnecessary social activities, flexible work system and quarantine policy, since the declaration of the COVID-19 epidemic on January 20, 2020. Another possible reason for the different rates of mental conditions may be due to the use of different clinical scales to assess psychological status. The occurrence of anxiety was not significantly increased during the COVID-19 pandemic among pregnant women, which may be related to the implementation of comprehensive prevention and control strategies. The COVID-19 epidemic had been effectively controlled over 30 days after the outbreak (29), which probably eased the fear of the COVID-19 epidemic.

Subgroup analysis found that psychological status was different among pregnancy trimesters. A greater likelihood of depression and higher levels of depressive symptoms were found in the first trimester, while greater levels of insomnia and psychological stress were reported in the third trimester. The patterns are consistent with previous studies conducted in Portugal (16) and Switzerland (30). One explanation may be that pregnant women went to hospital for regular antenatal care just once in the whole first trimester, and the outdoor activities decreased significantly as a prevention strategy during the pandemic. Thus, home quarantine and the resulted social isolation could have had negative impact on the mental health of pregnant women. Depression during early pregnancy is associated with depression and anxiety at late pregnancy and postpartum depression (17), and is associated with negative birth outcome and development of the infant (31, 32).

Pregnant women were more likely to suffer from clinically diagnosed insomnia in the third trimester. Previous research suggested that sleep quality decreases toward the end of the pregnancy (33, 34). The occurrence rate of psychological stress was similar to that (10.3%) in a U.S. study (27). The reason for significantly higher rates of psychological stress in the third trimester may be due to increased visits to hospital and worry about infection risk in childbirth, especially in a COVID-19 designated hospital.

Logistic regression analyses showed that previous psychological status was a risk factor for the current occurrence of the measured mental health problems, while more family support was a protective factor. Lack of social support predicted the occurrence of depression (35), consistent with our findings. Reduced support during pregnancy had a negative impact on maternal mental health outcomes (36). Family support may help with reducing mental problems for pregnant women during this COVID-19 pandemic.

## Limitations

There are some limitations that should be considered when interpreting the findings. First, this study was a cross-sectional epidemiologic study. Recall bias may happen when participants retrospectively rated their mental status before the COVID-19 outbreak. Second, structured diagnostic instruments were not applied to assess life-time psychiatric disorders. Third, only self-rated scales were applied to assess mental status. Fourth, some potential contributing factors to prenatal mental health such as marital disharmony and planned/unplanned pregnancy were not evaluated.

## Conclusion

To conclude, our data suggested that the COVID-19 pandemic had negative psychological impacts on pregnant women, particularly in the first and third trimesters and those who experienced mental issues before the outbreak. Early identification and intervention for mood symptoms in pregnant women particularly during a pandemic is important for the mother and fetus's physical and mental health. Mental health care from professional institutions should be implemented promptly on this special population.

## Data Availability Statement

The raw data supporting the conclusions of this article will be made available by the authors, without undue reservation.

## Ethics Statement

The studies involving human participants were reviewed and approved by Institutional Review Board of Guangzhou Women and Children Medical Care Center. The patients/participants provided their written informed consent to participate in this study.

## Author Contributions

KL conceptualized and designed the study and revised the manuscript. ZZ and RZ conducted the statistical analyses and drafted the manuscript. TL, PC, and YZ did the data collection. WL, GX, and K-FS provided critical comments. All authors contributed to the article and approved the submitted version.

## Conflict of Interest

The authors declare that the research was conducted in the absence of any commercial or financial relationships that could be construed as a potential conflict of interest.
